# SARS-CoV-2 spike S2-specific neutralizing antibodies

**DOI:** 10.1080/22221751.2023.2220582

**Published:** 2023-06-15

**Authors:** Chia-Jung Li, Shih-Chung Chang

**Affiliations:** a Department of Biochemical Science and Technology, College of Life Science, National Taiwan University, Taipei, Taiwan; b Center of Biotechnology, National Taiwan University, Taipei, Taiwan

**Keywords:** SARS-CoV-2, spike protein, S2 subunit, neutralizing antibody, antibody cocktail therapy

## Abstract

Since the onset of the coronavirus disease 2019 (COVID-19), numerous neutralizing antibodies (NAbs) against severe acute respiratory syndrome coronavirus 2 (SARS-CoV-2) have been developed and authorized for emergency use to control the pandemic. Most COVID-19 therapeutic NAbs prevent the S1 subunit of the SARS-CoV-2 spike (S) protein from binding to the human host receptor. However, the emergence of SARS-CoV-2 immune escape variants, which possess frequent mutations on the S1 subunit, may render current NAbs ineffective. In contrast, the relatively conserved S2 subunit of the S protein can elicit NAbs with broader neutralizing potency against various SARS-CoV-2 variants. In this review, the binding specificity and functional features of SARS-CoV-2 NAbs targeting different domains of the S2 subunit are collectively discussed. The knowledge learned from the investigation of the S2-specific NAbs provides insights and potential strategies for developing antibody cocktail therapy and next-generation coronavirus vaccine.

## Introduction

Coronaviruses belong to the *Coronavirinae* subfamily and are categorized into four genera based on genotypical and serological properties. Alphacoronaviruses (α-CoVs) and betacoronaviruses (β-CoV) mainly infect mammals, but deltacoronaviruses (δ-CoVs) and gammacoronaviruses (γ-CoVs) mainly infect birds [1,2]. Seven human coronaviruses (HCoVs) have been identified, including five β-CoVs (severe acute respiratory syndrome coronavirus 2 (SARS-CoV-2), severe acute respiratory syndrome coronavirus (SARS-CoV), Middle East respiratory syndrome coronavirus (MERS-CoV), HCoV-OC43, and HCoV-HKU1), and two α-CoVs (HCoV-NL63 and HCoV-229E) [1]. The common HCoVs have adapted to human hosts and contribute to 15%−30% of common cold with mild symptoms and upper-respiratory tract illness, though severe lower respiratory tract infections may sometimes happen in infants, elder people, and immunocompromised people [3,4]. However, the diseases caused by SARS-CoV-2, SARS-CoV, and MERS-CoV are more harmful for human health. The coronavirus disease 2019 (COVID-19) pandemic is caused by the highly pathogenic and transmissible coronavirus, SARS-CoV-2 [5]. Although SARS-CoV-2 has lower fatality rates compared to SARS-CoV and MERS-CoV [6], the continuous circulation of SARS-CoV-2 in humans leads to greater infection cases of COVID-19 worldwide. There have been 766,440,796 confirmed cases of COVID-19, including 6,932,591 deaths, reported to WHO, according to the WHO Coronavirus (COVID-19) Dashboard revealed on May 17, 2023. In the past 3 years, SARS-CoV-2 has evolved into several variants of concern (VOCs), including at least the Alpha, Beta, Gamma, Delta, Epsilon, and Omicron strains. Some SARS-CoV-2 VOCs possess mutations on the surface spike (S) proteins, rendering current therapeutic antibodies and vaccines ineffective. For example, the E484 mutation on the S proteins of SARS-CoV-2 Beta, Gamma, and Omicron variants makes viruses capable of escaping the neutralizing efficacy of several therapeutic antibodies and convalescent plasma [7]. Many VOCs have also acquired K417N/T and N501Y mutations in the ACE2 interaction surface of the S proteins [8–10].

**Figure 1.**
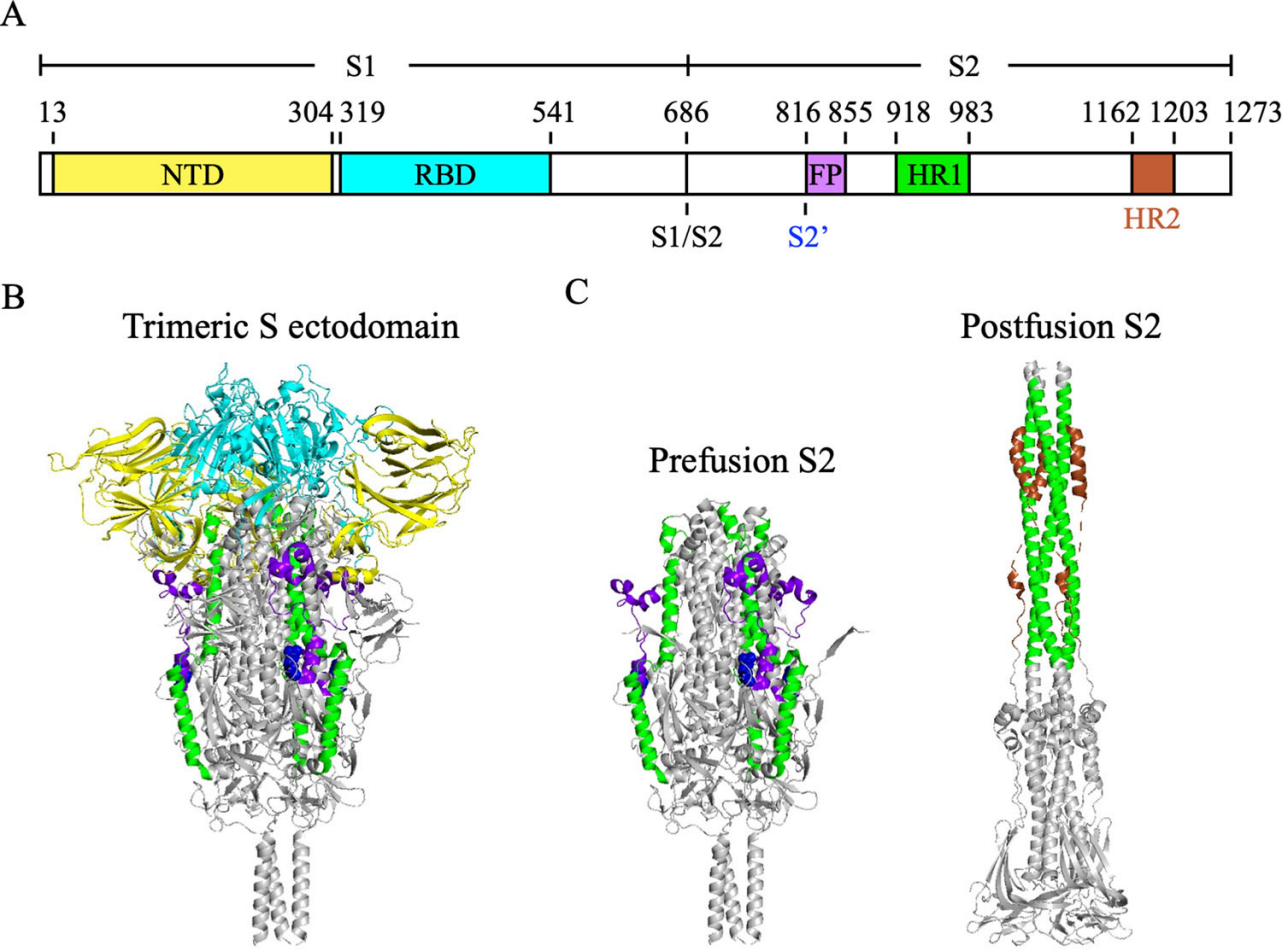
The molecular structure of SARS-CoV-2 S protein. (A) The schematic diagram of the domain structure of SARS-CoV-2 S protein. NTD, N-terminal domain (yellow). RBD, receptor-binding domain (cyan). S1/S2, S1/S2 furin cleavage site. S2’, S2’ cleavage site R815 (blue spheres). FP, fusion peptide (bright purple). HR1, heptad repeat 1 (green). HR2, heptad repeat 2 (brown). (B) The trimeric S protein ectodomain. (C) The prefusion (left) and postfusion (right) structures of the trimeric S2 subunits. HR2 was not seen in the crystal structure of the trimeric S protein ectodomain (PDB ID: 6XR8).

The core of SARS-CoV-2 consists of the nucleocapsid proteins and a single-stranded genomic RNA [11]. The viral core is encapsulated by phospholipid bilayers, which contain the S proteins, membrane proteins and envelope proteins [11], to form 80–120 nm spherical or pleomorphic particles. S proteins form homotrimers on the viral surface and are composed of two subunits, S1 and S2 (Figure 1A). At the junction of these two subunits is an S1/S2 furin cleavage site [12]. The S1 subunit (S_1-685_) contains the N-terminal domain (NTD, S_13-304_) and the receptor-binding domain (RBD, S_319-541_), which mediates the binding of virus to angiotensin-converting enzyme 2 (ACE2) receptor of the host cell [11,12]. The S2 subunit (S_686-1273_), which contains the fusion peptide (FP, S_816-855_), heptad repeat 1 (HR1, S_918-983_), and heptad repeat 2 (HR2, S_1163-1203_), is responsible for triggering the virus-host membrane fusion [11–13]. After SARS-CoV-2 S protein binding to the ACE2 receptor, the S1/S2 cleavage site is cleaved by host protease to shed the S1 subunit, resulting in the exposure of S2’ cleavage site on the S2 subunit [11,12]. The S2’ cleavage site is subsequently cleaved by transmembrane serine protease 2 (TMPRSS2) on the host cell membrane, leading to the exposure of the FP for initiating membrane fusion [11,12]. The FP inserts into the membrane of the host cell and then triggers the rearrangement of HR1 and HR2 into a postfusion six-helix bundle structure to pull together the membranes of the virus and the host cell [11,14].

Since S protein plays the key roles in mediating viral entry, it has become the main target of neutralizing antibodies (NAbs). In addition to antiviral drugs, such as nirmatrelvir/ritonavir (Paxlovid) and molnupiravir (Lagevrio), SARS-CoV-2 NAbs can also mitigate or completely inhibit viral infection, and therefore significantly reduce COVID-19 patient’s hospitalizations and disease severity [15–17]. SARS-CoV-2 NAbs can either be actively elicited by natural infection or vaccination, or be passively administered into individuals for prophylactic or therapeutic purposes [18]. SARS-CoV-2 NAbs may neutralize virus through inhibiting S1-ACE2 interaction or blocking the S2-mediated virus-host membrane fusion [18]. Besides reducing viral titres through Fab-mediated neutralization, NAbs can also inhibit viral infection through the Fc-mediated effector functions such as antibody-dependent cell-mediated cytotoxicity (ADCC), antibody-dependent cellular phagocytosis (ADCP), and complement-dependent cytotoxicity (CDC) [19]. Since the outbreak of COVID-19 pandemic, numerous NAbs against SARS-CoV-2, such as bebtelovimab, tixagevimab, and cilgavimab [20,21], have been developed and authorized for emergency use as COVID-19 therapeutics [18]. Most of SARS-CoV-2 NAbs target the S1 subunit, especially the RBD, to block virus binding to the host cell [3]. The RBD is also an important antigen of COVID-19 vaccines for the induction of NAbs [22]. Clinical studies revealed that several RBD-specific antibody therapies and vaccines lost their neutralizing potency due to the emergence of SARS-CoV-2 VOCs [18]. For example, REGEN-COV, an antibody cocktail therapy consisted of two RBD-specific NAbs, lost the neutralizing ability against the Omicron variant [23]. Another antibody therapy, sotrovimab, which also targets the RBD, lost the neutralizing potency against the Omicron BA.2 variant [24]. In response to the immune escape mutations of SARS-CoV-2, it is very important to develop NAbs with broader neutralizing spectrum. Compared to the S1 subunit, the amino acid sequences of the S2 subunit are relatively conserved among different SARS-CoV-2 VOCs [25]. Several studies have found that some antibodies targeting special S2 epitopes of the SARS-CoV-2 S protein tend to have broadly neutralizing activities [17,26–28]. In this review, the specificity and functionality of the S2-specific SARS-CoV-2 NAbs are discussed to provide insights and potential strategies for developing antibody therapy and next-generation coronavirus vaccine.

## NAbs targeting the S2 stem helix region

**Figure 2.**
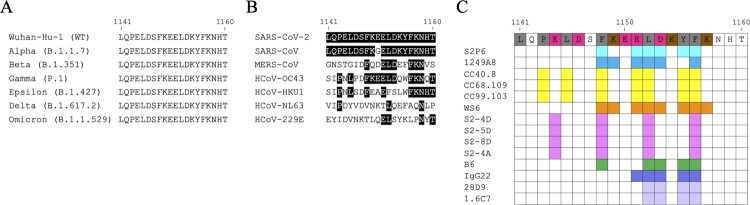
An immunodominant S2 peptide recognized by various S2 stem helix-specific antibodies. (A) Sequence alignment of the S2 stem helix regions (S_1141-1160_) of SARS-CoV-2 VOCs, including Wuhan-Hu-1, Alpha, Beta, Gamma, Epsilon, Delta, and Omicron strains. (B) Sequence alignment of the S2 stem helix regions of SARS-CoV-2, SARS-CoV, MERS-CoV, HCoV-OC43, HCoV-HKU1, HCoV-NL63, and HCoV-229E. S_1141-1160_ is shown as SARS-CoV-2 numbering. Residues identical to the sequence of SARS-CoV-2 S2 stem helix are marked with black backgrounds. (C) The key binding epitopes of the S2 stem helix-specific antibodies. Hydrophobic, negatively charged, and positively charged residues of S_1141-1160_ are coloured in grey, magenta, and brown, respectively. Residues recognized by S2P6 and 1249A8 that broadly neutralize SARS-CoV-2, SARS-CoV, and MERS-CoV are coloured in cyan and marine blue, respectively. Residues recognized by CC40.8, CC68.109, and CC99.103 that neutralize SARS-CoV-2 and SARS-CoV but not MERS-CoV are coloured yellow. Residues recognized by WS6 that neutralizes SARS-CoV-2 and SARS-CoV but not MERS-CoV are coloured in orange. Residues E1144, F1148, L1152, and F1156 recognized by S2-4D, S2-5D, S2-8D, and S2-4A that neutralize SARS-CoV-2 are coloured in pink. Residues recognized by B6, IgG22, 1.6C7, and 28D9 that only neutralize MERS-CoV but not SARS-CoV-2 are coloured in green, purple, and light purple, respectively.

When the trimeric S protein undergoes conformational change to turn the prefusion state (Figure 1B) into the postfusion state (Figure 1C), the S2 stem helix regions become extended to form a six-helix bundle [29–31]. Since the prefusion-to-postfusion structural transition of S protein is an essential step for virus-host membrane fusion, it is expected that antibodies targeting the S2 stem helix region may block SARS-CoV-2 infection [29]. It has been identified that the S2 stem helix is an immunogenic hotspot and contains immunodominant epitopes [32], which can elicit neutralizing antibodies [33]. Several S2 stem helix-specific NAbs were isolated from COVID-19 convalescent patients such as S2P6 [26], 1249A8 [17], CC40.8 [27], and CV3-25 [28] (Table 1), or generated by hybridoma technology such as WS6 [34], S2-4D [35], S2-5D [35], S2-8D [35], S2-4A [35], B6 [36], IgG22 [37], 28D9 [29], and 1.6C7 [29] (Table 1). However, it was reported that antibodies and memory B cells targeting the S2 stem helix region were rarely found in COVID-19 convalescent patients [38] or vaccinated individuals [39,40]. Notably, the S2 stem helix region within residues 1141–1160 of S protein [29] is conserved among various SARS-CoV-2 VOCs, including Alpha (B.1.1.7), Beta (B.1.351), Gamma (P.1), Epsilon (B.1.427), Delta (B.1.617.2), and Omicron (B.1.1.529) strains (Figure 2A), but is only partially conserved with MERS-CoV, HCoV-OC43, HCoV-HKU1, HCoV-229E and HCoV-NL63 (Figure 2B). Thus this region could be an ideal antigen for developing broadly neutralizing antibodies (bNAbs) [29,36,41].

**Table 1.** The SARS-CoV-2 S2 stem helix-specific neutralizing antibodies.

Antibody	Epitope region	Key binding residues	Source	Germline usage	Neutralizing potency	*In vivo* protection in animal models	Reference
S2P6	1148-1156	F1148, E1151, L1152, D1153, Y1155, F1156	COVID-19 patient	IGHV1-46	SARS-CoV-2	SARS-CoV-2 challenge in hamsters	[26]
					SARS-CoV		
				IGKV3-20	MERS-CoV		
					HCoV-OC43		
1249A8	1147-1158	F1148, E1151, L1152, D1153, F1156	COVID-19 patient	IGHV1-46	SARS-CoV-2	SARS-CoV-2 and SARS-CoV challenges in mice	[17,42]
					SARS-CoV		
				IGKV3-20			
					MERS-CoV		
CC40.8 C68.109 CC99.103	1142-1159	P1143, L1145, F1148, E1151, L1152, Y1155, F1156	COVID-19 patient	IGHV3-23	SARS-CoV-2	SARS-CoV-2 challenge in mice and hamsters	[27,43]
				IGKV3-20	SARS-CoV		
CV3-25	1149-1167	K1157, T1160, S1161, P1162, D1163, V1164, L1166	COVID-19 patient	IGHV5-51	SARS-CoV-2	SARS-CoV-2 challenge in mice	[28, 44, 46]
				IGKV1-12	SARS-CoV		
WS6	1143-1159	F1148, K1149, E1151, L1152, D1153, Y1155, F1156, K1157	Mouse immunized with S mRNA	IGHV1-5	SARS-CoV-2		[34]
				IGKV4-61	SARS-CoV		
S2-4D	1144-1156	E1144, F1148, L1152, F1156	Mice immunized with S2 protein		SARS-CoV-2		[35]
S2-5D							
S2-8D							
S2-4A							

### S2 stem helix-specific NAbs from COVID-19 convalescent patients

**Figure 3.**
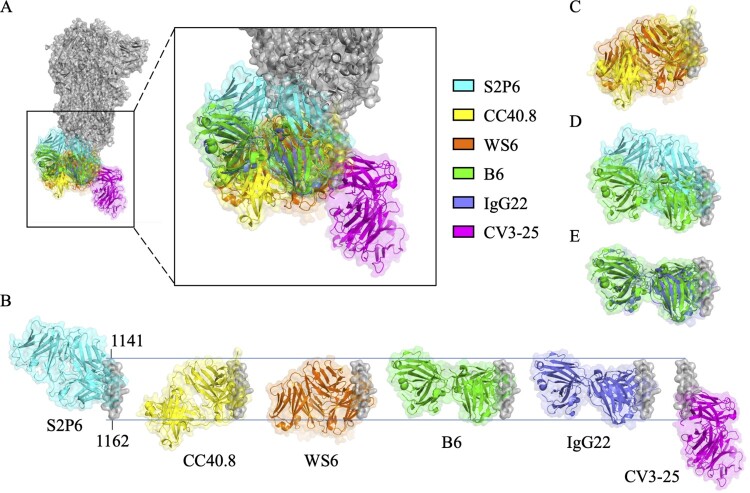
Superimposition of S2 stem helix-specific antibodies upon binding with their epitopes. (A) The Fabs of S2P6 (cyan, PDB: 7RNJ), CC40.8 (yellow, PDB: 7SJS), WS6 (orange, PDB: 7TCQ), B6 (green, PDB: 7M53), IgG22 (purple, PDB: 7S3N), and CV3-25 (magenta, PDB: 7RAQ) are superimposed with the prefusion S trimer (grey, PDB: 6XR8). (B) Alignment of the Fabs of S2P6, CC40.8, WS6, B6, IgG22, and CV3-25 upon binding with residues 1141-1162 of the S2 stem helix. Superimposition of the Fabs of S2P6 and B6 (C), CC40.8 and WS6 (D), or B6 and IgG22 (E) for comparison.

S2P6, an antibody isolated from a COVID-19 convalescent patient, can bind to S proteins of SARS-CoV-2, SARS-CoV, MERS-CoV, HCoV-OC43, and HCoV-HKU4 with high affinity, but bind to the S protein of HCoV-HKU1 with lower affinity. S2P6 can neutralize SARS-CoV, MERS-CoV, HCoV-OC43, and different SARS-CoV-2 VOCs including Wuhan-Hu-1, Alpha (B.1.1.7), Beta (B.1.351), Gamma (P.1), and Delta (B.1.617.1) variants [26]. However, it’s surprising that S2P6 failed to neutralize the Omicron variant [34]. S2P6 also provided *in vivo* protection in hamsters against SARS-CoV-2 infection by both Fab-mediated viral neutralization and Fc-mediated effector functions, including promoting ADCC and ADCP, instead of triggering CDC [26]. S2P6 targets the epitope sequence _1148_FKEELDKYF_1156_ and can bind to both of the prefusion and postfusion states of SARS-CoV-2 S protein. The structural analysis and alanine scanning experiment further showed that residues F_1148_, E_1151_, L_1152_, D_1153,_ Y_1155_, and F_1156_ are critical residues for S2P6 recognition [26] (Figure 2C and Table 1). It was proposed that S2P6 binding to the S2 stem helix might sterically interfere with S2 conformational change (Figure 3A and B), hence blocking virus-host membrane fusion [26]. Based on the binding specificity and sequence analysis, it is suggested that S2P6 might be arisen in response to previous HCoV-OC43 infection and acquired neutralization efficacy against SARS-CoV-2 through the somatic mutation [26].

1249A8, an antibody isolated from COVID-19 convalescent patient, could broadly recognize the S protein of various β-CoVs including SARS-CoV-2, SARS-CoV, MERS-CoV, HCoV-OC43, and HCoV-HKU1 (Table 1). 1249A8 also exhibited neutralizing potency against SARS-CoV-2, SARS-CoV, and MERS-CoV, and showed prophylactic and therapeutic activities against SARS-CoV and SARS-CoV-2, including Omicron variant, through the Fab-mediated neutralization and the Fc-mediated effector function [17]. The binding epitope of 1249A8, _1147_SFKEELDKYFKN_1158_, forms an amphipathic α-helix and is embedded in the groove formed by 1249A8 CDRs that interact with residues F_1148_, E_1151_, L_1152_, D_1153_ and F_1156_ (Figure 2C and Table 1). The structural analysis further suggested that 1249A8 mimicked the loop structure of the postfusion six-helix bundle to interact with the S2 stem helix and thus disrupted the formation of the postfusion structure [42]. Moreover, 1249A8 possessed high somatic hypermutation (SHM) and exhibited cross-reactivity with the S proteins of HCoV-OC43 and HKU1, suggesting that it might arise from a pre-existing memory B cell against seasonal CoV, and then acquired neutralization efficacy against SARS-CoV-2 infection [17].

CC40.8 was also isolated from a COVID-19 convalescent patient [27]. CC40.8 can neutralize SARS-CoV and different SARS-CoV-2 VOCs, including Wuhan-Hu-1, Alpha (B.1.1.7), Beta (B.1.351), Gamma (P.1), and Delta (B.1.617.2) strains through inhibiting virus-host membrane fusion [27]. CC40.8 exhibited *in vivo* protective efficacy and could reduce weight loss and lung viral titres in the SARS-CoV-2 viral challenging mouse and Syrian hamster animal models [27]. CC40.8 targets the epitope fragment _1142_QPELDSFKEELDKYFKNH_1159_ in the S2 stem helix region, which might be only partially exposed in the prefusion S trimer [27]. In addition, P_1143_, L_1145_, F_1148_, E_1151_, L_1152_, Y_1155_, and F_1156_ are important residues for CC40.8 binding [27] (Figure 2C and Table 1). Structural analysis further showed that the S_1142-1159_ epitope folds into a helical structure and was embedded mainly through hydrophobic interactions in a groove formed by CC40.8 heavy and light chain complementary determining regions (CDRs) (Figure 3A and B). It was proposed that CC40.8 bound to the intermediate state of the S2 subunit and disrupted the formation of the six-helix bundle in the postfusion S2 subunit, resulting in prohibited membrane fusion [27]. The conserved CC40.8 binding epitope was further used for the selection of NAbs from COVID-19 convalescent or vaccinated donors. The results of epitope mapping experiments indicated that these selected antibodies recognized a hydrophobic epitope, including F_1148_, L_1152_, and F_1156_, which might be poorly accessible in the prefusion S trimer. However, these selected antibodies still exhibited broadly neutralizing potency against β-CoVs and SARS-CoV-2 VOCs, including Alpha (B1.1.7), Beta (B.1.351), Gamma (P.1), Delta (B.1.617.2), and Omicron (B.1.1.529) strains. Notably, among the selected NAbs, CC68.109, and CC99.103 showed *in vivo* protection capability against SARS-CoV-2, SARS-CoV, and MERS-CoV in animal experiments [43]. Taken together, CC40.8 may access and bind to the epitope on the S protein which is undergoing prefusion-to-postfusion transition for blocking the formation of the six-helix bundle.

CV3-25, an antibody isolated from a COVID-19 convalescent patient [28], can bind to S proteins of SARS-CoV-2 and SARS-CoV, but not to MERS-CoV S protein [44]. CV3-25 can neutralize the authentic SARS-CoV-2 [45], Alpha (B.1.1.7), Beta (B.1.351), Gamma (P.1), Delta (B.1.61.72), and Omicron (B.1.1.529) variants, but also broadly neutralize SARS-CoV [28,45] by disrupting the membrane fusion step during virus infection [28]. Animal study showed that CV3-25 provided partial *in vivo* protection against SARS-CoV-2 infection in the K18-hACE2 transgenic mice through the Fab-mediated neutralization effector function [44]. Structural analysis showed that CV3-25 bound to a conserved epitope _1149_KEELDKYFKNHTSPDVDLG_1167_ [45] which was located at the base of the S2 stem helix. The results of epitope mapping experiments further showed that K_1157_, _1160_TSPDV_1164_, and L_1166_ are the key residues for CV3-25 binding [28] (Figure 2C and Table 1). Interestingly, the binding epitope of CV3-25 is more C-terminally extended than the binding epitopes of S2P6, 1249A8, and CC40.8 [46]. The different binding epitopes between CV3-25 and CC40.8 might lead to their distinct neutralizing potency against different CoVs. CV3-25 neutralized various SARS-CoV-2 VOCs and SARS-CoV but failed to inhibit MERS-CoV infection [28]. The CDRH1 and CDRH2 of CV3-25 bind to the N-terminal α-helix of the epitope (_1149_KEELDKYF_1156_) and the CDRH3 of CV3-25 interacts with the extended C-terminal random coil of the epitope (_1158_NHTSPDVDLG_1167_), leading to a unique bending structure [28,45,46]. The superimposition of the structure of the CV3-25 and its epitope to the prefusion S trimer (Figure 3A and B) showed that the binding of the CV3-25 light chain to the prefusion S trimer might clash with the prefusion-to-postfusion conformational transition, hence blocking the formation of the six-helix bundle [46].

### S2 stem helix-specific NAbs from immunized mice

The S2 stem helix-specific NAbs could also be produced by immunizing mice with SARS-CoV-2 S protein or S protein-based vaccine. WS6 is an S2 stem helix-specific mAb isolated from mice immunized with mRNA encoding the SARS-CoV-2 S protein [34]. WS6 recognized the S protein of SARS-CoV-2 and exhibited lower affinity against the S proteins of SARS-CoV, MERS-CoV, HCoV-OC43, and HCoV-HKU1. In addition to SARS-CoV-2 Alpha (B.1.1.7), Beta (B.1.351), Gamma (P.1), Delta (B.1.617.2), and Omicron (B.1.1.529) strains, WS6 also neutralized SARS-CoV by inhibiting S protein conformational change [34]. The structural analysis found that WS6 targeted the conserved epitope _1143_PELDSFKEELDKYFKNH_1159_ of SARS-CoV-2, which was a part of stem helix and embedded into a groove formed by all CDRs of WS6 heavy chains and light chains [34]. Epitope mapping experiments indicated that F_1148_, L_1152_, Y_1155_, and F_1156_ are critical binding sites targeted by WS6, and K_1149_, E_1151_, D_1153_, and K_1157_ are also involved in antibody recognition [34] (Figure 2C and Table 1). It is also worth mentioning that the Fabs of CC40.8 and WS6 have very similar structures while binding with their epitopes (Figure 3C) although they were derived from different antibody production systems.

S2-4D, S2-5D, S2-8D, and S2-4A are also S2 stem helix-specific mAbs generated by the conventional hybridoma technology from mice immunized with SARS-CoV-2 S2 subunit [35]. S2-4D, S2-5D, S2-8D, and S2-4A exhibited cross-reactivity against the S proteins of SARS-CoV-2, SARS-CoV, and MERS-CoV. S2-4D, S2-5D, S2-8D, and S2-4A inhibited SARS-CoV-2 infection through inhibiting S protein-mediated membrane fusion and possessed broadly neutralizing activity against SARS-CoV-2 Alpha (B.1.1.7), Beta (B.1.351), Gamma (P.1), Delta (B.1.617.2), Epsilon (B.1.429) [35], and Omicron (unpublished data) variants. Epitope mapping analysis showed that the key residues E_1144_, F_1148_, L_1152_, and F_1156_ of the epitope peptide S_1144-1156_ were required for S2-4D, S2-5D, S2-8D, and S2-4A binding. Notably, S2-4D, S2-5D, S2-8D, and S2-4A are a group of unique mAbs that can specifically recognize E_1144_ in the S2 stem helix region. More importantly, antisera from mice immunized with S_1127-1167_ showed potent SARS-CoV-2 neutralizing activities and the COVID-19 convalescent sera also contain specific antibodies targeting S_1127-1167_, suggesting that this region is a potential vaccine candidate [35].

### S2 stem helix-specific Abs can bind SARS-CoV-2 S protein but lacking neutralizing activity

B6 was a pan-β-CoV antibody isolated from mice receiving the prime immunization of the prefusion-stabilized MERS-CoV S ectodomain trimer and a subsequent booster of the prefusion-stabilized SARS-CoV S ectodomain trimer [36]. B6 can tightly bind to the epitope peptides of MERS-CoV S_1230–1244_, HCoV-OC43 S_1232–1246_, HCoV-HKU4 S_1231–1245_, SARS-CoV S_1129–1143_, and SARS-CoV-2 S_1147–1161_. Notably, B6 failed to neutralize SARS-CoV-2 and SARS-CoV [36]. The crystal structure of the complex of B6 Fab and its epitope peptide _1230_DFQDELDEFFKNVST_1244_ of MERS-CoV S protein showed that it folded as an amphipathic ɑ-helix and docked its hydrophobic face, composed of F_1231_, L_1235_, F_1238_ and F_1239_ (corresponding to F_1148_, L_1152_, F_1155_, and F_1156_ of SARS-CoV-2 S protein) (Figure 2C, into a hydrophobic groove formed by B6 heavy and light chains [36], Figure 3A and B). The superimposition of the Fabs of S2P6 and B6 showed that they bound to the similar epitope with very different orientations (Figure 3D), suggesting that the epitope accessibility and the correct binding orientation should be the key factors for the determination of the neutralizing activity of the S2 stem helix-specific antibody.

Another S2 stem helix-specific murine antibody, IgG22, was isolated from mice immunized with the stabilized S2 subunit of MERS-CoV S protein. IgG22 can bind to the S proteins of MERS-CoV, SARS-CoV, SARS-CoV-2, but only showed neutralizing potency against MERS-CoV. Similar with B6, IgG22 binds to MERS-CoV S protein through recognizing the key residues of L_1235_, F_1238_, and F_1239_ (corresponding to L_1152_, Y_1155_, and F_1156_ of SARS-CoV-2 S protein) [37] (Figure 2C). Interestingly, superimposition of the Fabs of B6 and IgG22 showed that they have very similar structures upon binding with their epitopes (Figure 3E). This evidence also supports the criteria that the epitope accessibility and the binding orientation of the S2 stem helix-specific antibody may affect the efficacy of the NAbs although they recognize the same conserved regions found in various CoVs.

## NAbs targeting the S2’cleavage site or FP

**Figure 4.**
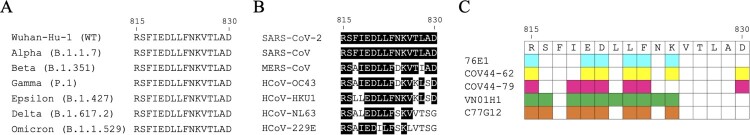
Sequence alignment of the partial FP residues recognized by S2’/FP-specific antibodies. (A) Sequence alignment of the partial S2’/FP regions (S_815-830_) of SARS-CoV-2 VOCs, including Wuhan-Hu-1, Alpha, Beta, Gamma, Epsilon, Delta, and Omicron strains. (B) Sequence alignment of the partial S2’/FP regions of SARS-CoV-2, SARS-CoV, MERS-CoV, HCoV-OC43, HCoV-HKU1, HCoV-NL63, and HCoV-229E. S_815-830_ is shown as SARS-CoV-2 numbering. Residues identical to the S2’/FP sequence of SARS-CoV-2 are marked with black backgrounds. (C) The key residues recognized by 76E1, COV44-62, COV44-79, and VN01H1 that can neutralize both of α- and β-CoVs are coloured in cyan, yellow, magenta, and green, respectively. The key residues recognized by C77G12 that can only neutralize β-CoVs are coloured in orange.

Proteolytic cleavage at the S2’ site of S protein and outstretch of FP are necessary for the S protein-mediated membrane fusion. The sequence of FP is 100% conserved among SARS-CoV-2 VOCs (Figure 4A) and remains conserved in α-CoVs and β-CoVs (Figure 4B). Antibodies targeting FP exhibited broad binding ability to S proteins of α-CoVs and β-CoVs, including SARS-CoV-2 VOCs [47]. In addition, FP was identified as an immunodominant epitope [32,48–50]. Previous studies showed that the depletion of antibodies targeting FP reduced the SARS-CoV-2 neutralizing ability of the COVID-19 positive sera [51]. Therefore, it is expected that the FP-specific antibody may have neutralizing activity against CoVs.

76E1 is an FP-specific antibody isolated from a COVID-19 convalescent patient [52]. 76E1 can cross-react to S proteins of some α-CoVs (e.g. HCoV-229E and HCoV-NL63) and β-CoVs (e.g. SARS-CoV-2, SARS-CoV, MERS-CoV, HCoV-OC43, and HCoV-HKU1). 76E1 exhibited neutralizing activity against SARS-CoV-2 wild type, Alpha (B.1.1.7), Beta (B.1.351), Gamma (P.1), Delta (B.1.617.1), and Omicron (B.1.1.529) variants [52] (Table 2). Furthermore, 76E1 provided *in vivo* protection against SARS-CoV-2 and HCoV-OC43 challenges in both prophylactic and therapeutic murine models [52]. 76E1 can bind to S_809-833_ which contains the highly conserved S2’ cleavage site R_815_ and the N-terminal region of FP. Epitope mapping experiments identified that R_815_, E_819_, and F_823_ were important binding residues of 76E1, and D_820_, L_822_, and K_825_ were also involved in antibody recognition (Figure 4C). Since FP is partially buried in the prefusion state of S trimer (Figure 1B) and R_815_ is shielded by the N-terminal loop (residues 804-806) of FP, 76E1 is expected to be incapable of binding to the prefusion S trimer. Structural analysis showed that binding of 76E1 to the S2’ cleavage site R_815_ may inhibit S2’ cleavage and the subsequent cascade of S2 conformational rearrangement, implying that FP is a promising target for neutralizing antibody or vaccine candidate [52]. However, it is controversial that two FP-specific antibodies 555D6 and 125C1 isolated from COVID-19 convalescent patients did not exhibit neutralizing activity against SARS-CoV-2 although they can also bind to the FP region (S_809-823_) [52]. The different neutralizing potency between 555D6, 125C1, and 76E1 revealed that only certain FP-specific antibody exhibited SARS-CoV-2 neutralizing activity [52]. Hence, the immunization strategy for specifically eliciting 76E1-like NAbs is a challenge for developing pan-coronavirus vaccines. As mentioned previously, FP will become exposed after S protein binding to ACE2 and the cleavage of the S2’ site is the key step to facilitate S2 subunit undergoing the prefusion-to-postfusion conformational transition. Based on these observations, it is predicted that ACE2 binding to S protein is the pivotal step to trigger the exposure of FP for 76E1 binding. Therefore, the cocktail therapy combining 76E1 with ACE2 was applied to examine the enhancement of the neutralizing potency against SARS-CoV-2. The results showed that the combination of 76E1 with ACE2 exhibited synergistic effect in preventing SARS-CoV-2 infection [52]. Interestingly, an RBD-specific antibody CB6 also exhibited synergistic effect with 76E1 in neutralizing SARS-CoV-2, implying that CB6 is an ACE2-mimicking antibody [52]. In addition, P2C-1F11, a previously reported antibody with ACE2 mimicry feature [53], also synergistically enhances the neutralizing potency of 76E1 to combat SARS-CoV-2. These data suggest a potential combination therapy by using 76E1 and ACE2 or ACE2-mimicking antibody to synergistically neutralize SARS-CoV-2.

**Table 2.** The SARS-CoV-2 S2’/FP-specific neutralizing antibodies.

Antibody	Epitope region	Key binding residues	Source	Germline usage	Neutralizing potency	*In vivo* protection in animal models	Reference
76E1	809-833	R815, E819, D820, L822, F823, K825	COVID-19 patient	IGHV3-43	β-CoVs SARS-CoV-2	SARS-CoV-2 and HCoV-OC43 challenges in mice	[52]
					SARS-CoV		
					MERS-CoV		
					HCoV-OC43		
				IGLV2-8			
					α-CoVs		
					HCoV-229E		
					HCoV-NL63		
COV44-62	815-823	K814, R815, E819, D820, L822, F823, K825, D830	COVID-19 patient	IGHV1-2	β-CoVs	SARS-CoV-2 challenge in hamsters	[54]
					SARS-CoV-2		
				IGLV2-8			
					SARS-CoV		
					MERS-CoV		
					HCoV-OC43		
					α-CoVs		
					HCoV-229E		
					HCoV-NL63		
COV44-79	815-823	R815, I818, E819, D820, L822, F823, D830	COVID-19 patient	IGHV3-30	β-CoVs	SARS-CoV-2 challenge in hamsters	[54]
					SARS-CoV-2		
					SARS-CoV		
					HCoV-OC43		
				IGKV1-12	α-CoVs		
					HCoV-229E		
					HCoV-NL63		
VN01H1	811-825	R815, S816, I818, E819, D820, L821, L822, F823, N824, K825	COVID-19 patient		β-CoVs	SARS-CoV-2 challenge in hamsters	[56]
					SARS-CoV-2		
					SARS-CoV		
					MERS-CoV		
					α-CoVs		
					HCoV-229E		
					HCoV-NL63		
C77G12	811-825	R815, S816, I818, E819, D820, L821, L822, F823, N824, K825	COVID-19 patient		β-CoVs	SARS-CoV-2 challenge in hamsters	[56]
					SARS-CoV-2		
					SARS-CoV		
					MERS-CoV		

COV44-62 and COV44-79 are also FP-specific SARS-CoV-2 NAbs isolated from COVID-19 convalescent patients. COV44-62 and COV44-79 can inhibit virus-host membrane fusion [54] and neutralize SARS-CoV, HCoV-OC43, HCoV-NL63, HCoV-229E, and SARS-CoV-2 VOCs including Omicron BA.2 and BA.5 subvariants (Table 2). COV44-62 could further neutralize MERS-CoV. In addition, COV44-62 and COV44-79 possessed *in vivo* protection ability against SARS-CoV-2 challenge in the Syrian hamster model [54]. COV44-62 and COV44-79 target _815_RSFIEDLLF_823_ within the N-terminal region of FP, which adopts a helical structure and is partially solvent-exposed in the prefusion S protein. Binding analysis showed that COV44-62 and COV44-79 have higher affinity to S2 subunit than to prefusion S protein, suggesting that its binding epitope might be shielded by the S1 subunit. Interestingly, epitope mapping experiments indicated that COV44-62 and COV44-79 bind to the same face of the FP helix with different angles. K_814_, R_815_, E_819_, D_820_, L_822_, F_823_, K_825_, and D_830_ are key residues for COV44-62 binding, but the critical residues for COV-44-79 binding are partially different, including R_815_, I_818_, E_819_, D_820_, L_822_, F_823_, and D_830_ (Figure 4C and Table 2). Although FP was predicted as an immunodominant epitope and was important for serum-neutralizing potency, the serological analysis found that FP-specific antibodies were only elicited in some COVID-19 patients and were rarely found in vaccinated donors [55]. This realistic situation might be due to the less exposure of COV44-62 and COV44-79 binding epitopes which are shielded by the S1 subunit and therefore rarely presented to B cells during natural infection or vaccination.

VN01H1 and C77G12 are also FP-specific antibodies isolated from COVID-19 convalescent patients [56]. VN01H1 can neutralize β-CoVs (e.g. SARS-CoV-2, SARS-CoV, MERS-CoV) as well as α-CoVs (e.g. HCoV-NL63 and HCoV-229E), whereas C77G12 can only neutralize β-CoVs [56]. Both antibodies could further neutralize SARS-CoV-2 Omicron BA.1 and BA.2 variants and provide *in vivo* protection against SARS-CoV-2 infection in hamsters [56]. VN01H1 and C77G12 target _811_KPSKRSFIEDLLFNK_825_ which contains the S2’ cleavage site R_815_ as well as the N-terminal region of FP. Structural analysis showed that key residues recognized by VN01H1 and C77G12 are R_815_, S_816_, I_818,_ E_819,_ D_820_, L_821_, L_822_, F_823_, N_824_, and K_825_ (Figure 4C and Table 2) [56]. VN01H1 and C77G12 likely bind to R_815_ through electrostatic interaction for blocking S2’ cleavage by TMPRSS2 and therefore preventing the activation of virus-host membrane fusion. The binding epitopes of VN01H1 and C77G12 are buried inside the core region of prefusion S trimer and become exposed after ACE2 binding. In addition, the neutralizing ability of VN01H1 and C77G12 could be improved by engineering the full-length antibodies into the single chain variable fragment (scFv) [56], suggesting that the smaller size of antibodies can increase its accessibility for binding to the epitopes in FP. Furthermore, the ACE2-mimicking antibody S2E12 could enhance VN01H1 and C77G12 binding to S protein and synergistically increase neutralizing effect [56]. Based on these findings, it is hypothesized that ACE2 or ACE2-mimicking antibodies promote exposure of the S2’cleavage site which is then more accessible for the FP-specific antibody binding.

**Figure 5.**
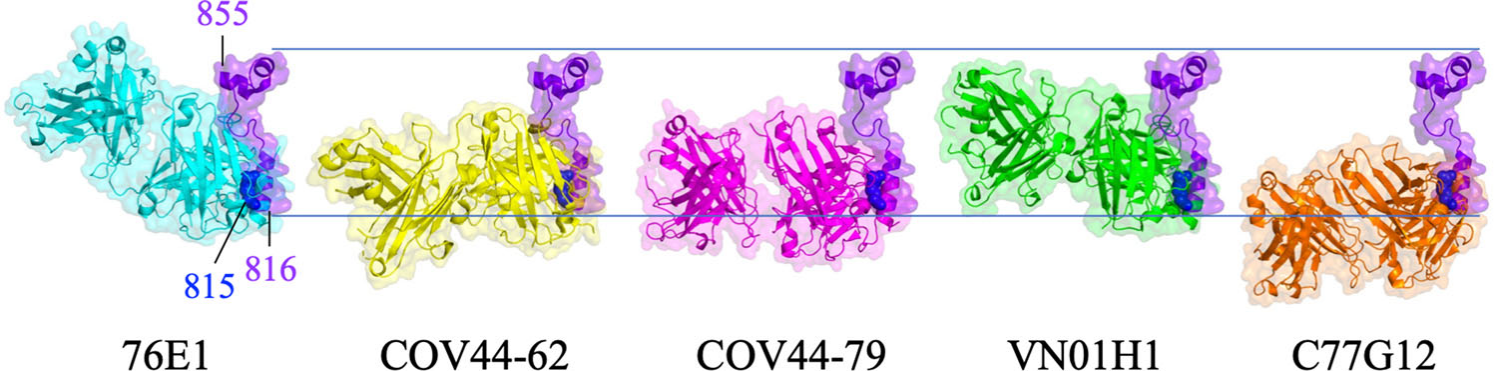
Superimposition of S2’/FP-specific antibodies upon binding with their epitopes. The Fabs of 76E1 (cyan, PDB: 7X9E), COV44-62 (yellow, PDB: 8D36), COV44-79 (magenta, PDB: 8DAO), VN01H1 (green, PDB: 7SKZ), and C77G12 (orange, PDB: 7U0A) are superimposed with the FP (bright purple) of SARS-CoV-2 S protein. N’ and C’ stand for the N- and C-termini of FP.

In brief, the FP-specific NAbs mentioned above (76E1, COV44-62, COV44-79, VN01H1, and C77G12) that target the S2’ cleavage site and the N-terminal region of FP (Figures 4–5C) were mainly isolated from COVID-19 patients. These antibodies inhibit viral infection possibly through blocking S2’ cleavage by TMPRSS2 or endosomal cathepsins, hence preventing S2 from structural rearrangement for virus-host membrane fusion. However, only a small fraction of FP-specific antibodies possessed broadly neutralizing ability [56]. Thus the strategy for eliciting NAbs using the S2’ cleavage site or FP as the immunogen should be carefully designed to efficiently induce neutralizing antibodies.

## NAb targeting HR2

The postfusion state of the trimeric S2 is formed by a central HR1 triple-helix bundle and additionally surrounded by HR2 [57] (Figure 1C). Ek1 is a peptide derived from HR2 which can bind to HR1 for blocking HR1–HR2 interaction and neutralizing SARS-CoV-2 through inhibiting virus-host membrane fusion [58]. It is reported that an *in silico* designed peptide inhibitor based on the HR2 sequences can specifically bind to the HR1 domain of SARS-CoV-2 S protein and prevent the S2 subunit from forming the pre-hairpin conformation, thus potentially blocking SARS-CoV-2 infection [59]. A previous study also found that antibodies targeting HR1 and HR2 of SARS-CoV S protein can neutralize SARS-CoV [60]. However, the NAb targeting HR1 or HR2 of SARS-CoV-2 S protein was poorly investigated.

**Figure 6.**
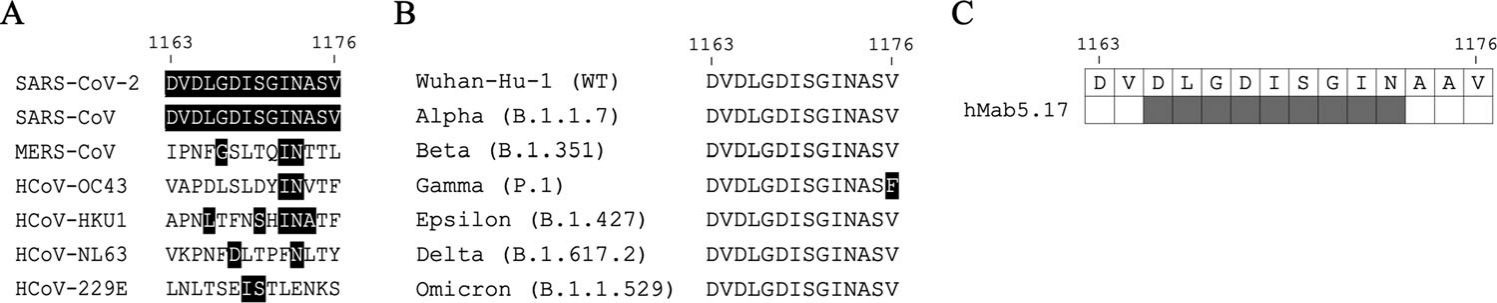
Sequence alignment of the partial HR2 residues recognized by hMab5.17. (A) Sequence alignment of the partial HR2 residues of SARS-CoV-2, SARS-CoV, MERS-CoV, HCoV-OC43, HCoV-HKU1, HCoV-NL63, and HCoV-229E. S_1163-1176_ is shown as SARS-CoV-2 numbering. Residues identical to the sequence of SARS-CoV-2 HR2 are marked with black backgrounds. (B) Sequence alignment of the partial HR2 residues (S_1163-1176_) of SARS-CoV-2 VOCs, including Wuhan-Hu-1, Alpha, Beta, Gamma, Epsilon, Delta, and Omicron strains. F1176 in Gamma strain is marked with black background. (C) The binding epitope of hMab5.17 on the SARS-CoV-2 S protein is coloured in grey.

hMab5.17 is a humanized antibody derived from a murine antibody Mab5, which was elicited by immunization of BALB/c mice with SARS-CoV S protein [57,61]. hMab5.17 can bind to the HR2 domains of SARS-CoV-2 and SARS-CoV S proteins but not the HR2 domains of other β-CoVs, probably due to the different levels of HR2 sequence similarity (Figure 6A). In addition, hMab5.17 can neutralize SARS-CoV and SARS-CoV-2 VOCs including Alpha (B.1.1.7), Beta (B.1.351), Gamma (P.1), and Delta (B.1.617.2) variants [57]. Notably, though the S protein of Gamma strain has the V1176F mutation in the highly conserved HR2 domain (Figure 6B), the neutralizing activity of Mab5.17 against Gamma strain remains effective [57]. hMab5.17 also provided *in vivo* protection against the challenges of SARS-CoV-2 WT and Delta strains in Syrian hamsters and reduced weight loss, viral titre, and pathological changes. hMab5.17 targets the highly conserved epitope _1165_DLGDISGIN_1173_ [57] (Figure 6C), which is located at the N-terminal end of the HR2 domain and is well-exposed in both prefusion and postfusion conformational states of S protein. The binding epitope of hMab5.17 folds into an extended N-terminal loop and a C-terminal one-turn helix, and wraps around the central HR1 triple-helix bundle [57]. Sequence alignment further reveals that this epitope is only conserved in SARS-CoV-like viruses but not in other CoVs (Figure 6A), explaining the binding and neutralizing specificity of Mab5.17 against SARS-CoV-2 and SARS-CoV [57]. Since the HR2 regions are almost 100% conserved among SARS-CoV-2 VOCs (except the Gamma strain containing the V1176F mutation) (Figure 6B), HR2 has the potential for elicitation of SARS-CoV-2 bNAbs.

## Antibody cocktail therapy

High mutation rates of SARS-CoV-2 led to resistance against antibody treatment. Therefore, cocktail therapy might be used to minimize immune escape of SARS-CoV-2, as the virus required simultaneous mutations at multiple epitopes to avoid neutralization by NAbs [62]. Several cocktail therapies against SARS-CoV-2 had been developed and approved by FDA for clinical use. REGN-COV2 was composed of two RBD-specific antibodies imdevimab and casirivimab, and could effectively neutralize SARS-CoV-2 WT, Alpha, Gamma, and Delta variants. However, REGN-COV2 lost neutralizing ability against the Omicron variant, indicating that the antibody cocktail therapy needs to be further explored once an NAb evasion strain has emerged [63]. Evusheld^TM^, is a combination of two human monoclonal antibodies, tixagevimab and cilgavimab, targeting the surface S protein of SARS-CoV-2. Tixagevimab + cilgavimab combination demonstrated to neutralize SARS-CoV-2 pre-omicron strains. However, recently emerged Omicron sub-lineages have exhibited the ability to escape this cocktail’s activity. Although Evusheld^TM^ could have maintained some benefit for COVID-19 prophylaxis and treatment in immunosuppressed and vulnerable populations that cannot be vaccinated (for safety reasons or immune diseases), the utility of this cocktail could have compromised [64].

It has been reported that a combination of S2 stem helix-specific NAb 1249A8 and S1-specific NAb 1213H7 through direct respiratory administration could synergistically protect mice from SARS-CoV-2 WT, SARS-CoV-2 Delta variant, and SARS-CoV infections [17]. Another research also found that a combination of S2 stem helix-specific NAb CV3-25 and RBD-specific antibody CV3-1 could effectively protect mice from SARS-CoV-2 infection [44]. Besides S2 stem helix-specific antibodies, FP-specific antibodies were also applied in cocktail therapy. As described previously, the combination of the FP-specific antibody 76E1 and ACE2 or ACE2-mimicking antibodies (CB6 or P2C-1F11) exhibited synergistic effect in preventing SARS-CoV-2 infection [52]. It’s also found that the combination of the FP-specific antibody VN01H1 or C77G12 and ACE2-mimicking antibody S2E12 possessed synergistic neutralizing effect [56]. Therefore, it is suggested that the combination of S1-specific and S2-specific antibodies for COVID-19 treatment is a promising strategy to prevent the emergence of the immune escape strains.

## Vaccine development

Since the outbreak of COVID-19, numerous vaccines have been developed and several have been authorized for emergency use [65–67]. The emerged SARS-CoV-2 variants, especially those possessing RBD mutations, exhibited resistance to vaccine-stimulated NAbs [68]. Furthermore, the SARS-CoV-2 BA.1 and BA2 neutralizing titres of the sera derived from BNT1162b2-vaccinated donors were 20-fold lower than the titres against the D614G strain [69]. Mutations in RBD or NTD of the S protein often caused antibody evasion [70]. Previous studies have identified or predicted S_812-829_, S_1148-1159_, and S_1256-1273_ as antigenic hot areas in the S2 subunit [32,49,71–74]. S_812-829_-specific IgGs were strongly elicited in both COVID-19 patients and mRNA-vaccinated donor [75], demonstrating its high immunogenicity. S_818-835_-specific antibodies were significantly correlated with the neutralizing IC_50_ values and depletion of these antibodies reduced the serum-neutralizing potency for inhibiting SARS-CoV-2 pseudovirus infection [51]. S_811-830_-specific antibodies elicited by previous HCoV infection could mitigate the severity of COVID-19 [32]. Furthermore, it was found that antibodies against S_1148-1159_ possessed neutralizing activity [74]. S_1148-1159_-specific IgG responses in the COVID-19 non-survivor group were significantly lower than that in the survivor group, suggesting the protective roles of the S2 stem helix-specific antibodies [71]. Another study also identified that S_1146-1165_ was an immunodominant epitope and antibodies against this peptide partially contributed to the SARS-CoV-2 neutralizing potency of the COVID-19-convalescent patient sera [76]. These findings suggested that FP and S2 stem helix-specific antibodies really played important roles in preventing SARS-CoV-2 infection. Additionally, the S2 cross-reactive antibodies isolated from COVID-19 patients exhibited broader ranges of binding ability against various SARS-CoV-2 VOCs than antibodies against the S1 subunit [41]. To avoid immune escape and specifically elicit NAbs for combating SARS-CoV-2 WT and various VOCs, the relatively conserved FP and S2 stem helix seem to be better vaccine candidates [77].

It has been reported that the pre-existing immunity against endemic HCoVs may also contribute to modulate immune responses in the SARS-CoV-2-unexposed subjects for mitigating COVID-19 severity [78,79]. It was proposed that some S2 stem helix-specific NAbs, such as CC40.8 and S2P6, might be primed by previously endemic CoV and then be re-activated by SASR-CoV-2 infection for developing more broadly reactive antibodies [28]. The high SHM levels of most FP-specific antibodies also suggested the same hypothesis [56]. It was also found that the B cells of COVID-19 patients have increased SHM and higher affinity against the S proteins of HCoVs than that of the pre-pandemic individuals, indicating that SARS-CoV-2 exposure might train the HCoV-induced B cells to produce more effective SARS-CoV-2 NAbs [41]. Immunogenetic analysis of the S2 stem helix-specific antibody sequences showed strong enrichment of IGHV1-46 (63%) and IGHV3-23 (22%) germline gene families [43]. For light chain gene usage, a strong enrichment of IGKV3–20 (47%) and IGLV1–51 (16%) germline gene families was found [43]. Of note, S2P6 and 1249A8 are encoded by IGHV1-46 and IGKV3-20. CC40.8 is encoded by IGHV3-23 and IGKV3-20. Some unique IgG germline gene repertoires served as the foundation for consisting of the S2 stem helix-cross reactive NAbs.

## Conclusion

NAbs can be used for COVID-19 treatment [18–21], but the emergence of various SARS-CoV-2 VOCs which possessed immune escape mutations in the RBD may render current NAbs and vaccines ineffective [22–24]. The relatively conserved S2 subunit of the SARS-CoV-2 S protein might be a better COVID-19 vaccine candidate for eliciting bNAbs against various SARS-CoV-2 VOCs. The S2 stem helix region contains immunogenic epitopes [29,32,33,74,80], which could induce potent NAbs against SARS-CoV-2 infection and mitigate COVID-19 severity [29,36,41] (Figure 2, Figure 4C, and Figure 6C). Interestingly, most of the S2 stem helix-specific NAbs bind to the N-terminus of the amphipathic stem helix which docks into a hydrophobic groove formed by the NAbs [26, 27, 36, 37, 42]. Some SARS-CoV-2 NAbs also recognize the acidic residues in the S2 stem helix region. For instance, S2-4D, S2-5D, S2-8D, and S2-4A can recognize the hydrophilic residue E_1144_ [35], and CV3-25 recognizes D_1153_ on the hydrophilic face of the S2 stem helix [17].

Despite the broadly neutralizing activity, it has been observed that the neutralizing titres of the S2 stem helix-specific antibodies were relatively lower than those of the S1- or RBD-targeting antibodies. CV3-25 neutralized various SARS-CoV-2 strains with IC50 values ranging from 0.05 to 0.2 μg/mL [46]. In contrast, the RBD-specific antibody CV3-1 neutralized various SARS-CoV-2 strains with IC50 values ranging from 0.004 to 0.014 μg/mL [46]. S2P6 neutralized the authentic SARS-CoV-2 with the IC50 value of 1.67 μg/mL, but the RBD-specific antibody S309 exhibited the IC50 value of 0.04 μg/mL [26]. 1249A8 neutralized the SARS-CoV-2 Omicron virus with the IC50 value of 2.4 μg/mL, but the S1-specific neutralizing antibody 1213H7 exhibited the IC50 value of 0.06 μg/mL [17]. These findings revealed that the potency metrics of the S2 stem helix-specific antibodies are 10–40 folds lower than those of S1- or RBD-targeting antibodies. Therefore, the improvement of the potency of the S2 stem helix-specific antibodies remains a challenge to be conquered in the future.

The FP within residues 816–855 of S protein is conserved among α-CoVs and β-CoVs. Antibodies targeting this region showed broad cross-activation against S proteins of α-CoVs and β-CoVs, including various SARS-CoV-2 VOCs. NAbs targeting S2’ cleavage site/FP inhibit viral infection through blocking the S2’ cleavage process and the subsequent S2 rearrangement for membrane fusion [52, 4,56]. Structural analysis showed that S2’ cleavage site/FP is buried in the interior core of the trimeric S protein [52,54] and becomes exposed after binding of S protein to the ACE2 receptor. A combination of S2’ cleavage site/FP-specific NAbs and ACE2 protein or ACE2-mimicking antibodies as a cocktail therapy exhibited synergistic effect in preventing SARS-CoV-2 infection [52,56].

Several studies have developed HR1-specific peptides that can inhibit SARS-CoV-2 infection by blocking the formation of the six-helix bundle [57–59]. The HR2-specific mAb hMAb5.17 can neutralize SARS-CoV-2 and SARS-CoV [57]. However, the NAbs against HR1 or HR2 were rarely found, probably due to the poor immunogenicity or the inaccessibility of the region in the prefusion state of the trimeric S protein.

The FP and the S2 stem helix region were identified as immunogenic hot areas [32,49,71–74] which can induce NAbs for inhibiting SARS-CoV-2 infection and mitigating COVID-19 severity [51,76]. Nevertheless, the antibodies binding to the same S2-neutralizing epitope with different orientations may exhibit distinct neutralizing activities. Moreover, designing vaccines to elicit NAbs encoded by the specific germline and to mimic the re-exposure of different HCoV antigens is also important for developing pan-HCoV vaccines [28,41,56]. S2 stem helix-specific NAbs (e.g. S2P6 [26], CC40.8 [27], and 1249A8 [17]) and S2’ cleavage site/FP-specific NAbs (e.g. COV44-62 [54], VN01H1 [56], and C77G12 [56]) with high SHM may be primed by previous endemic HCoVs and then be reactivated for specific maturation during SARS-CoV-2 infection. Thus, based on the knowledge learned from the studies of S2-specific NAbs, the challenges in developing vaccines for COVID-19 or other CoVs should be further investigated in the future.

## References

[1] Brix TH, Hegedus L. Severe acute respiratory syndrome coronavirus-2 (SARS-CoV-2) infection and thyroid disease. An update. Curr Opin Endocrinol Diabetes Obes. 2021;28:525–532. doi:10.1097/MED.0000000000000654 34224435 PMC8452244

[2] Paim FC, Bowman AS, Miller L, et al. Epidemiology of deltacoronaviruses (delta-CoV) and gammacoronaviruses (gamma-CoV) in wild birds in the United States. Viruses. 2019;11. 10.3390/v11100897 PMC6832366 31561462

[3] Barnes CO, West AP, Huey-Tubman KE, et al. Structures of human antibodies bound to SARS-CoV-2 spike reveal common epitopes and recurrent features of antibodies. Cell. 2020;182:828–842. doi:10.1016/j.cell.2020.06.025 32645326 PMC7311918

[4] Mesel-Lemoine M, Millet J, Vidalain PO, et al. A human coronavirus responsible for the common cold massively kills dendritic cells but not monocytes. J Virol. 2012;86:7577–7587. doi:10.1128/JVI.00269-12 22553325 PMC3416289

[5] Shrestha LB, Tedla N, Bull RA. Broadly-neutralizing antibodies against emerging SARS-CoV-2 variants. Front Immunol. 2021;12 752003. doi:10.3389/fimmu.2021.752003 34646276 PMC8502962

[6] Yi Y, Lagniton PNP, Ye S, et al. COVID-19: what has been learned and to be learned about the novel coronavirus disease. Int J Biol Sci. 2020;16:1753–1766. doi:10.7150/ijbs.45134 32226295 PMC7098028

[7] Yang WT, Huang WH, Liao TL, et al. SARS-CoV-2 E484K mutation narrative review: epidemiology, immune escape, clinical implications, and future considerations. Infect Drug Resist. 2022;15:373–385. doi:10.2147/IDR.S344099 35140483 PMC8820839

[8] Li Q, Nie J, Wu J, et al. SARS-CoV-2 501Y.V2 variants lack higher infectivity but do have immune escape. Cell. 2021;184:2362–2371. doi:10.1016/j.cell.2021.02.042 33735608 PMC7901273

[9] Zhou D, Dejnirattisai W, Supasa P, et al. Evidence of escape of SARS-CoV-2 variant B.1.351 from natural and vaccine-induced sera. Cell. 2021;184:2348–2361. doi:10.1016/j.cell.2021.02.037 33730597 PMC7901269

[10] Garcia-Beltran WF, Lam EC, St Denis K, et al. Multiple SARS-CoV-2 variants escape neutralization by vaccine-induced humoral immunity. Cell. 2021;184:2372–2383. doi:10.1016/j.cell.2021.03.013 33743213 PMC7953441

[11] Lamers MM, Haagmans BL. SARS-CoV-2 pathogenesis. Nat Rev Microbiol. 2022;20:270–284. doi:10.1038/s41579-022-00713-0 35354968

[12] Harrison AG, Lin T, Wang P. Mechanisms of SARS-CoV-2 transmission and pathogenesis. Trends Immunol. 2020;41:1100–1115. doi:10.1016/j.it.2020.10.004 33132005 PMC7556779

[13] Wrapp D, Wang N, Corbett KS, et al. Cryo-EM structure of the 2019-nCoV spike in the prefusion conformation. Science. 2020;367:1260–1263. doi:10.1126/science.abb2507 32075877 PMC7164637

[14] Li X, Yuan H, Li X, et al. Spike protein mediated membrane fusion during SARS-CoV-2 infection. J Med Virol. 2023;95:e28212. 36224449 10.1002/jmv.28212 PMC9874878

[15] Akinosoglou K, Schinas G, Gogos C. Oral antiviral treatment for COVID-19: a comprehensive review on nirmatrelvir/ritonavir. Viruses. 2022;14. 10.3390/v14112540 PMC9696049 36423149

[16] Wong CKH, Au ICH, Lau KTK, et al. Real-world effectiveness of early molnupiravir or nirmatrelvir-ritonavir in hospitalised patients with COVID-19 without supplemental oxygen requirement on admission during Hong Kong's omicron BA.2 wave: a retrospective cohort study. Lancet Infect Dis. 2022;22:1681–1693. doi:10.1016/S1473-3099(22)00507-2 36029795 PMC9401976

[17] Piepenbrink MS, Park JG, Deshpande A, et al. Potent universal beta-coronavirus therapeutic activity mediated by direct respiratory administration of a spike S2 domain-specific human neutralizing monoclonal antibody. PLoS Pathog. 2022;18:e1010691. doi:10.1371/journal.ppat.1010691 35862475 PMC9302814

[18] Li D, Sempowski GD, Saunders KO, et al. SARS-CoV-2 neutralizing antibodies for COVID-19 prevention and treatment. Annu Rev Med. 2022;73:1–16. doi:10.1146/annurev-med-042420-113838 34428080

[19] Tso FY, Lidenge SJ, Poppe LK, et al. Presence of antibody-dependent cellular cytotoxicity (ADCC) against SARS-CoV-2 in COVID-19 plasma. PLoS One. 2021;16:e0247640. 33661923 10.1371/journal.pone.0247640 PMC7932539

[20] McCreary EK, Kip KE, Collins K, et al. Evaluation of bebtelovimab for treatment of COVID-19 during the SARS-CoV-2 omicron variant era. Open Forum Infect Dis. 2022;9:ofac517. doi:10.1093/ofid/ofac517 36324319 PMC9619560

[21] Keam SJ. Tixagevimab + Cilgavimab: first approval. Drugs. 2022;82:1001–1010. doi:10.1007/s40265-022-01731-1 35727563 PMC9211051

[22] Dickey TH, Tang WK, Butler B, et al. Design of the SARS-CoV-2 RBD vaccine antigen improves neutralizing antibody response. Sci Adv. 2022;8:eabq8276. doi:10.1126/sciadv.abq8276 36103542 PMC9473567

[23] Flahault A, Touchard J, Pere H, et al. Breakthrough omicron COVID-19 infections in patients receiving the REGEN-Cov antibody combination. Kidney Int. 2022;101:824–825. doi:10.1016/j.kint.2022.01.016 35157894 PMC8837470

[24] Takashita E, Yamayoshi S, Simon V, et al. Efficacy of antibodies and antiviral drugs against omicron BA.2.12.1, BA.4, and BA.5 subvariants. N Engl J Med. 2022;387:468–470. doi:10.1056/NEJMc2207519 35857646 PMC9342381

[25] Olukitibi TA, Ao Z, Warner B, et al. Significance of conserved regions in coronavirus spike protein for developing a novel vaccine against SARS-CoV-2 infection. Vaccines (Basel). 2023;11. 10.3390/vaccines11030545 PMC10056353 36992129

[26] Pinto D, Sauer MM, Czudnochowski N, et al. Broad betacoronavirus neutralization by a stem helix-specific human antibody. Science. 2021;373:1109–1116. doi:10.1126/science.abj3321 34344823 PMC9268357

[27] Zhou P, Yuan M, Song G, et al. A human antibody reveals a conserved site on beta-coronavirus spike proteins and confers protection against SARS-CoV-2 infection. Sci Transl Med. 2022;14:eabi9215. doi:10.1126/scitranslmed.abi9215 35133175 PMC8939767

[28] Hurlburt NK, Homad LJ, Sinha I, et al. Structural definition of a pan-sarbecovirus neutralizing epitope on the spike S2 subunit. Commun Biol. 2022;5:342. doi:10.1038/s42003-022-03262-7 35411021 PMC9001700

[29] Wang C, van Haperen R, Gutierrez-Alvarez J, et al. A conserved immunogenic and vulnerable site on the coronavirus spike protein delineated by cross-reactive monoclonal antibodies. Nat Commun. 2021;12:1715. doi:10.1038/s41467-021-21968-w 33731724 PMC7969777

[30] Walls AC, Tortorici MA, Snijder J, et al. Tectonic conformational changes of a coronavirus spike glycoprotein promote membrane fusion. Proc Natl Acad Sci U S A. 2017;114:11157–11162. doi:10.1073/pnas.1708727114 29073020 PMC5651768

[31] Cai Y, Zhang J, Xiao T, et al. Distinct conformational states of SARS-CoV-2 spike protein. Science. 2020;369:1586–1592. doi:10.1126/science.abd4251 32694201 PMC7464562

[32] Shrock E, Fujimura E, Kula T, et al. Viral epitope profiling of COVID-19 patients reveals cross-reactivity and correlates of severity. Science. 2020;370. doi:10.1126/science.abd4250 PMC7857405 32994364

[33] Ladner JT, Henson SN, Boyle AS, et al. Epitope-resolved profiling of the SARS-CoV-2 antibody response identifies cross-reactivity with endemic human coronaviruses. Cell Rep Med. 2021;2:100189. doi:10.1016/j.xcrm.2020.100189 33495758 PMC7816965

[34] Shi W, Wang L, Zhou T, et al. Vaccine-elicited murine antibody WS6 neutralizes diverse beta-coronaviruses by recognizing a helical stem supersite of vulnerability. Structure. 2022;30:1233–1244. doi:10.1016/j.str.2022.06.004 35841885 PMC9284671

[35] Li CJ, Chao TL, Chang TY, et al. Neutralizing monoclonal antibodies inhibit SARS-CoV-2 infection through blocking membrane fusion. Microbiol Spectr. 2022;10:e0181421. 35293796 10.1128/spectrum.01814-21 PMC9045258

[36] Sauer MM, Tortorici MA, Park YJ, et al. Structural basis for broad coronavirus neutralization. Nat Struct Mol Biol. 2021;28:478–486. doi:10.1038/s41594-021-00596-4 33981021

[37] Hsieh CL, Werner AP, Leist SR, et al. Stabilized coronavirus spike stem elicits a broadly protective antibody. Cell Rep. 2021;37:109929. doi:10.1016/j.celrep.2021.109929 34710354 PMC8519809

[38] Dacon C, Peng L, Lin TH, et al. Rare, convergent antibodies targeting the stem helix broadly neutralize diverse betacoronaviruses. Cell Host Microbe. 2023;31:97–111. doi:10.1016/j.chom.2022.10.010 36347257 PMC9639329

[39] Seow J, Graham C, Hallett SR, et al. Chadox1 nCoV-19 vaccine elicits monoclonal antibodies with cross-neutralizing activity against SARS-CoV-2 viral variants. Cell Rep. 2022;39:110757. doi:10.1016/j.celrep.2022.110757 35477023 PMC9010245

[40] Amanat F, Thapa M, Lei T, et al. SARS-CoV-2 mRNA vaccination induces functionally diverse antibodies to NTD, RBD, and S2. Cell. 2021;184:3936–3948. doi:10.1016/j.cell.2021.06.005 34192529 PMC8185186

[41] Song G, He WT, Callaghan S, et al. Cross-reactive serum and memory B-cell responses to spike protein in SARS-CoV-2 and endemic coronavirus infection. Nat Commun. 2021;12:2938. doi:10.1038/s41467-021-23074-3 34011939 PMC8134462

[42] Deshpande A, Schormann N, Piepenbrink MS, et al. Structure and epitope of a neutralizing monoclonal antibody that targets the stem helix of β coronaviruses. bioRxiv. 2022;2022.09.14.507947. 10.1111/febs.16777 PMC10330828 37014961

[43] Zhou P, Song G, Liu H, et al. Broadly neutralizing anti-S2 antibodies protect against all three human betacoronaviruses that cause deadly disease. Immunity. 2023;56:669–686. doi:10.1016/j.immuni.2023.02.005. PMC9933850 36889306

[44] Ullah I, Prevost J, Ladinsky MS, et al. Live imaging of SARS-CoV-2 infection in mice reveals that neutralizing antibodies require Fc function for optimal efficacy. Immunity. 2021;54:2143–2158. doi:10.1016/j.immuni.2021.08.015 34453881 PMC8372518

[45] Jennewein MF, MacCamy AJ, Akins NR, et al. Isolation and characterization of cross-neutralizing coronavirus antibodies from COVID-19+ subjects. Cell Rep. 2021;36:109353. doi:10.1016/j.celrep.2021.109353 34237283 PMC8216847

[46] Li W, Chen Y, Prevost J, et al. Structural basis and mode of action for two broadly neutralizing antibodies against SARS-CoV-2 emerging variants of concern. Cell Rep. 2022;38:110210. doi:10.1016/j.celrep.2021.110210 34971573 PMC8673750

[47] Vanderheijden N, Stevaert A, Xie J, et al. Functional analysis of human and feline coronavirus cross-reactive antibodies directed against the SARS-CoV-2 fusion peptide. Front Immunol. 2021;12:790415. doi:10.3389/fimmu.2021.790415 35069571 PMC8766817

[48] Morgenlander WR, Henson SN, Monaco DR, et al. Antibody responses to endemic coronaviruses modulate COVID-19 convalescent plasma functionality. J Clin Invest. 2021;131. doi:10.1172/JCI146927 PMC8011893 33571169

[49] Wang H, Wu X, Zhang X, et al. SARS-CoV-2 proteome microarray for mapping COVID-19 antibody interactions at amino acid resolution. ACS Cent Sci. 2020;6:2238–2249. doi:10.1021/acscentsci.0c00742 33372199 PMC7586461

[50] Ng KW, Faulkner N, Cornish GH, et al. Preexisting and de novo humoral immunity to SARS-CoV-2 in humans. Science. 2020;370:1339–1343. doi:10.1126/science.abe1107 33159009 PMC7857411

[51] Poh CM, Carissimo G, Wang B, et al. Two linear epitopes on the SARS-CoV-2 spike protein that elicit neutralising antibodies in COVID-19 patients. Nat Commun. 2020;11:2806. doi:10.1038/s41467-020-16638-2 32483236 PMC7264175

[52] Sun X, Yi C, Zhu Y, et al. Neutralization mechanism of a human antibody with pan-coronavirus reactivity including SARS-CoV-2. Nat Microbiol. 2022;7:1063–1074. doi:10.1038/s41564-022-01155-3 35773398

[53] Ge J, Wang R, Ju B, et al. Antibody neutralization of SARS-CoV-2 through ACE2 receptor mimicry. Nat Commun. 2021;12:250. doi:10.1038/s41467-020-20501-9 33431856 PMC7801515

[54] Dacon C, Tucker C, Peng L, et al. Broadly neutralizing antibodies target the coronavirus fusion peptide. Science. 2022;377:728–735. doi:10.1126/science.abq3773 35857439 PMC9348754

[55] Wang Z, Schmidt F, Weisblum Y, et al. mRNA vaccine-elicited antibodies to SARS-CoV-2 and circulating variants. Nature. 2021;592:616–622. doi:10.1038/s41586-021-03324-6 33567448 PMC8503938

[56] Low JS, Jerak J, Tortorici MA, et al. ACE2-binding exposes the SARS-CoV-2 fusion peptide to broadly neutralizing coronavirus antibodies. Science. 2022;377:735–742. doi:10.1126/science.abq2679 35857703 PMC9348755

[57] Wu WL, Chiang CY, Lai SC, et al. Monoclonal antibody targeting the conserved region of the SARS-CoV-2 spike protein to overcome viral variants. JCI Insight. 2022;7. 10.1172/jci.insight.157597 PMC9089791 35290246

[58] Xia S, Zhu Y, Liu M, et al. Fusion mechanism of 2019-nCoV and fusion inhibitors targeting HR1 domain in spike protein. Cell Mol Immunol; 2020(17):765–767. 10.1038/s41423-020-0374-2 PMC7075278 32047258

[59] Ling R, Dai Y, Huang B, et al. *In silico* design of antiviral peptides targeting the spike protein of SARS-CoV-2. Peptides. 2020;130:170328. doi:10.1016/j.peptides.2020.170328 32380200 PMC7198429

[60] Elshabrawy HA, Coughlin MM, Baker SC, et al. Human monoclonal antibodies against highly conserved HR1 and HR2 domains of the SARS-CoV spike protein are more broadly neutralizing. PLoS One. 2012;7:e50366. doi:10.1371/journal.pone.0050366 23185609 PMC3503966

[61] Lai SC, Chong PC, Yeh CT, et al. Characterization of neutralizing monoclonal antibodies recognizing a 15-residues epitope on the spike protein HR2 region of severe acute respiratory syndrome coronavirus (SARS-CoV). J Biomed Sci. 2005;12:711–727. doi:10.1007/s11373-005-9004-3 16132115 PMC7089214

[62] Baum A, Fulton BO, Wloga E, et al. Antibody cocktail to SARS-CoV-2 spike protein prevents rapid mutational escape seen with individual antibodies. Science. 2020;369:1014–1018. doi:10.1126/science.abd0831 32540904 PMC7299283

[63] Nguyen H, Lan PD, Nissley DA, et al. Cocktail of REGN antibodies binds more strongly to SARS-CoV-2 than its components, but the omicron variant reduces its neutralizing ability. J Phys Chem B. 2022;126:2812–2823. doi:10.1021/acs.jpcb.2c00708 35403431 PMC9016775

[64] Convertino I, Ferraro S, Cappello E, et al. Tixagevimab + cilgavimab against SARS-CoV-2: the preclinical and clinical development and real-world evidence. Expert Opin Drug Discov. 2023;18:231–245. doi:10.1080/17460441.2023.2170348 36649625

[65] Polack FP, Thomas SJ, Kitchin N, et al. Safety and efficacy of the BNT162b2 mRNA COVID-19 vaccine. N Engl J Med. 2020;383:2603–2615. doi:10.1056/NEJMoa2034577 33301246 PMC7745181

[66] Baden LR, El Sahly HM, Essink B, et al. Efficacy and safety of the mRNA-1273 SARS-CoV-2 vaccine. N Engl J Med. 2021;384:403–416. doi:10.1056/NEJMoa2035389 33378609 PMC7787219

[67] Krammer F. SARS-CoV-2 vaccines in development. Nature. 2020;586:516–527. doi:10.1038/s41586-020-2798-3 32967006

[68] Krause PR, Fleming TR, Longini IM, et al. SARS-CoV-2 variants and vaccines. N Engl J Med. 2021;385:179–186. doi:10.1056/NEJMsr2105280 34161052 PMC8262623

[69] Zhou H, Dcosta BM, Landau NR, et al. Resistance of SARS-CoV-2 omicron BA.1 and BA.2 variants to vaccine-elicited sera and therapeutic monoclonal antibodies. Viruses. 2022: 14. 35746806 10.3390/v14061334 PMC9228817

[70] Harvey WT, Carabelli AM, Jackson B, et al. SARS-CoV-2 variants, spike mutations and immune escape. Nat Rev Microbiol. 2021;19:409–424. doi:10.1038/s41579-021-00573-0 34075212 PMC8167834

[71] Li Y, Ma ML, Lei Q, et al. Linear epitope landscape of the SARS-CoV-2 spike protein constructed from 1,051 COVID-19 patients. Cell Rep. 2021;34:108915. doi:10.1016/j.celrep.2021.108915 33761319 PMC7953450

[72] Zamecnik CR, Rajan JV, Yamauchi KA, et al. Rescan, a multiplex diagnostic pipeline, pans human sera for SARS-CoV-2 antigens. Cell Rep Med. 2020;1:100123. doi:10.1016/j.xcrm.2020.100123 32995758 PMC7513813

[73] Ahmed SF, Quadeer AA, McKay MR. COVIDep: a web-based platform for real-time reporting of vaccine target recommendations for SARS-CoV-2. Nat Protoc. 2020;15:2141–2142. doi:10.1038/s41596-020-0358-9 32555466 PMC7299140

[74] Li Y, Lai DY, Zhang HN, et al. Linear epitopes of SARS-CoV-2 spike protein elicit neutralizing antibodies in COVID-19 patients. Cell Mol Immunol. 2020;17:1095–1097. doi:10.1038/s41423-020-00523-5 32895485 PMC7475724

[75] Yin D, Ling S, Tian X, et al. A single dose SARS-CoV-2 simulating particle vaccine induces potent neutralizing activities. bioRxiv. 2020. 2020.05.14.093054.

[76] Yi Z, Ling Y, Zhang X, et al. Functional mapping of B-cell linear epitopes of SARS-CoV-2 in COVID-19 convalescent population. Emerg Microbes Infect. 2020;9:1988–1996. doi:10.1080/22221751.2020.1815591 32844713 PMC7534331

[77] Liu H, Wilson IA. Protective neutralizing epitopes in SARS-CoV-2. Immunol Rev. 2022;310:76–92. doi:10.1111/imr.13084 35599305 PMC9348472

[78] Guthmiller JJ, Wilson PC. Remembering seasonal coronaviruses. Science. 2020;370:1272–1273. doi:10.1126/science.abf4860 33303605

[79] Ng KT, Mohd-Ismail NK, Tan YJ. Spike S2 subunit: the dark horse in the race for prophylactic and therapeutic interventions against SARS-CoV-2. Vaccines (Basel). 2021;9. 10.3390/vaccines9020178 PMC7923282 33672450

[80] Chen Y, Zhao X, Zhou H, et al. Broadly neutralizing antibodies to SARS-CoV-2 and other human coronaviruses. Nat Rev Immunol. 2022: 1–11. 36168054 10.1038/s41577-022-00784-3 PMC9514166

